# Clinical Significance of Perinephric Fluid Collection in Patients with Renal Colic and Urolithiasis: A Retrospective Analysis

**DOI:** 10.3390/jcm13206118

**Published:** 2024-10-14

**Authors:** Stefano Moretto, Ugo Gradilone, Giovanni Costanzi Porrini, Marco Montesi, Antonio Cretì, Pierluigi Russo, Filippo Marino, Nazario Foschi, Marcello Covino, Francesco Pinto, Mauro Ragonese

**Affiliations:** 1 Department of Urology, Fondazione Policlinico Universitario Agostino Gemelli IRCCS, Università Cattolica del Sacro Cuore, 00136 Roma, Italy; gradilone.ug@gmail.com (U.G.); mauroragonese@yahoo.it (M.R.); 2 Department of Urology, Humanitas Clinical and Research Center, Rozzano, 20089 Milano, Italy; 3 Department of Biomedical Sciences, Humanitas University, 20072 Milano, Italy; 4 Department of Emergency Medicine, Fondazione Policlinico Universitario Agostino Gemelli IRCCS, Università Cattolica del Sacro Cuore, 00136 Roma, Italy

**Keywords:** perinephric fluid, urolithiasis, ultrasound, renal colic, hydronephrosis

## Abstract

**Background**: Perinephric fluid is commonly identified in patients with renal colic and urolithiasis, especially in cases associated with hydronephrosis. However, its clinical relevance, particularly its impact on treatment decisions and prognosis, is not well established. **Methods**: This retrospective, single-center study included adult patients who presented to the emergency department (ED) with suspected renal colic between January 2021 and October 2023. Patients underwent ultrasound evaluations, which were analyzed for perinephric fluid, hydronephrosis, stone size, and stone location. Data on patient demographics, laboratory results, and clinical outcomes, including the need for urological interventions, were also collected and analyzed. Multivariate logistic regression was used to assess factors associated with perinephric fluid presence. **Results**: Of the 509 patients included, 200 (39.3%) had perinephric fluid. Hydronephrosis was significantly associated with perinephric fluid (OR: 4.14, *p* = 0.007), as were stones located in the proximal (OR: 3.06, *p* = 0.003) and distal ureter (OR: 2.31, *p* = 0.018). However, sonographic perinephric fluid did not significantly affect the likelihood of urological intervention, in-hospital complications, sepsis, acute kidney injury (AKI), acute kidney disease (AKD), and prolonged hospital stay. **Conclusions**: Perinephric fluid is a common finding in patients with renal colic and urolithiasis, particularly in cases involving hydronephrosis and ureteral stones. However, despite its prevalence, sonographic perinephric fluid was not significantly associated with the need for urological intervention, longer hospital stays, or worse clinical outcomes. Further prospective studies are required to clarify its clinical implications fully.

## 1. Introduction

Urolithiasis is a highly common condition, with its prevalence varying from 1 to 20% of the population, depending on geographical regions [1]. Since the early 1990s, the Emergency Department (ED) use by patients presenting with upper tract urolithiasis in the USA has almost doubled to 340 per 100,000 persons despite total ED visits only increasing by 16% [2]. An upper tract stone may completely block the collecting system and compromise adequate urine flow, causing hydronephrosis, which is linked to a variety of complications, including infection, ureteral stricture development, and renal failure [3,4,5]. Such cases are regarded as urologic emergencies and need urgent urinary tract decompression, while definitive removal of the stone should not be completed until the patient’s condition improves [6].

When a diagnosis of nephrolithiasis is clinically suspected, imaging of the kidneys, ureters, and bladder should be performed to support the diagnosis of a stone [1]. Non-contrast computed tomography scan (NCCT) has become the standard of care for diagnosing acute flank pain. However, ultrasound (US) is recommended as the primary diagnostic imaging tool [1], as it is considered an acceptable alternative to NCCT [7,8]. US can directly identify stones located in the kidneys and at the pyelo–ureteral and vesico–ureteric junctions, though it often fails to detect ureteral calculi. Regarding sonographic secondary signs, hydronephrosis is well established in the setting of point of care ultrasound (POCUS) in the ED as an important sign to assess stone size and to predict thirty-day outcomes and the risk of ureteral stricture [3,4,9,10,11]. Perinephric fluid may be an overlooked sonographic finding associated with acute ureteral obstruction, especially when accompanied by hydronephrosis [10], but has also been reported in the setting of procedural complications, pseudoaneurysms, neoplasms, abscess formation, renal laceration with hematoma, and perirenal lymphangiomatosis. There are two main theories in the literature regarding the pathophysiology of perinephric fluid. According to one theory, one of the kidney’s responses to increased pressure in the ureter is the absorption of urine, which then infiltrates the perinephric space along the bridging septa [12]. The other theory suggests that the perinephric fluid collection results from a urinary leak secondary to forniceal rupture. In acute ureteral stone obstruction, a sharp rise in intrapelvic pressure can cause the collecting system to rupture at its weakest point—the fornices [13,14]. To the best of our knowledge, only two studies have shown the correlation between perinephric fluid accumulation and the management of lithiasis [15,16]. The aim of this study is to evaluate the clinical significance of sonographic perinephric fluid collection in patients with renal colic and evidence of urolithiasis.

## 2. Materials and Methods

### 2.1. Study Design 

After the approval of the local Ethics Committee of Fondazione Policlinico Universitario A. Gemelli IRCCS, we performed an observational, retrospective, single-center study based on the electronic medical record review of consecutive patients using a prospectively maintained database. 

### 2.2. Patient Recruitment

The recruitment period spanned from January 2021 to October 2023. The inclusion criteria were adult patients (age ≥ 18 years) who presented to the ED with suspected renal colic and underwent US evaluation with a urolithiasis finding. The sonographic evaluation was conducted by experienced radiologists who assessed the presence or the absence of the following parameters: perinephric fluid collection, hydronephrosis, stone size, and stone position. Patients were excluded if they had a final diagnosis other than nephrolithiasis (e.g., pyelonephritis or urinary tract obstruction unrelated to stone disease), if they were pregnant, or if they had a solitary kidney. 

### 2.3. Data Collection

**Figure 1:**
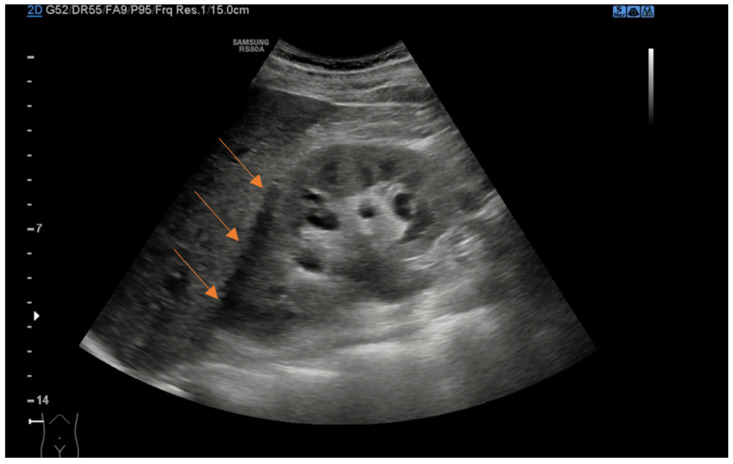
Coronal ultrasound view of right kidney demonstrating anechoic perinephric fluid (arrows).

We retrospectively collected data from patient charts, which included baseline demographic and clinical characteristics such as age, sex, body mass index (BMI), comorbidities, previous history of kidney stones or renal colic, initial presentation symptoms, and the presence of fever. Clinical and laboratory data at the time of ED presentation were also recorded, including the diameter of the kidney stones in millimeters, stone location, presence of perinephric fluid, presence of hydronephrosis, and laboratory findings such as creatinine level and leukocytosis, which provided information on renal function and potential infection. Using a curvilinear probe, experienced US radiologists examined the urinary system with a Samsung RS80A US machine. Perinephric fluid was defined as any anechoic stripe within the perirenal space bordered by the renal parenchyma and Gerota’s and Zuckerkandl’s fasciae, not including the anterior or posterior pararenal spaces, or the subcapsular space (Figure 1) [17]. The presence of hydronephrosis was defined as renal pelvic/calyx dilatation. The severity of hydronephrosis was defined according to the renal pelvic diameter (mm): 0–10 mild, 11–20 moderate, and >20 severe. Patients with hydronephrosis upon US without evidence of the cause of obstruction underwent a CT scan to evaluate the presence of a ureteral stone not visible with the US. Additionally, treatment-related data were gathered, including whether patients were discharged home or required hospitalization from the ED, the specific type of intervention performed (such as medical expulsive therapy, ureteral stent placement, nephrostomy tube insertion, or retrograde intrarenal surgery), and the length of hospital stay. For patients with favorable clinical conditions and ureteral stones less than 10 mm, we opted for conservative management and initiated medical expulsive therapy. These patients were discharged from the ED with instructions to return if they experienced recurrent renal colic that did not respond to analgesic therapy or if they developed a fever. Otherwise, they were scheduled for a follow-up urology visit 2 weeks after ED discharge, which included US and bladder exams. Patients with recurrent colic, not responsive to analgesic therapy, or patients with signs and symptoms of infections or compromised renal function were generally treated with immediate surgical intervention, which involved stent or nephrostomy placement, or ureteroscopy when feasible.

### 2.4. Outcome Measures

The primary outcome of this study was to determine the association between sonographic perinephric fluid collection and clinical outcomes in patients presenting with renal colic and confirmed urolithiasis. This included evaluating the correlation between perinephric fluid collection and the likelihood of requiring urological interventions, such as ureteral stent placement, nephrostomy tube insertion, or retrograde intrarenal surgery. A sub-analysis was conducted within this cohort to assess the association between perinephric fluid presence and clinical outcomes, focusing on in-hospital complications, the incidence of sepsis, and the use of antibiotics, analgesics, and anti-inflammatory drugs, as well as the development of acute kidney injury (AKI) and acute kidney disease (AKD). Additionally, we evaluated whether patients with perinephric fluid collection had longer hospital stays than those without.

AKD was defined according to the 2020 Kidney Disease Improving Global Outcomes (KDIGO) consensus conference criteria as postoperative AKI occurrence, decrease in estimated glomerular filtration rate (eGFR) ≥ 35%, or increase in serum creatinine (SCr) level ≥ 50% anytime between postoperative day 1 and day 59 [18]. Postoperative AKI was defined and staged according to the KDIGO criteria [19]. Specifically, AKI was defined as SCr increase ≥0.3 mg/dL or >50%. eGFR was calculated according to the CKD-EPI creatinine equation (2021), and the CKD category was calculated according to the National Kidney Foundation (NKF) classification [20]. 

The secondary outcome evaluated the association between sonographic perinephric fluid and potential clinical predictors, including hydronephrosis, kidney stone size and location, fever, creatinine levels, and leukocytosis in the ER.

### 2.5. Statistical Analysis

We analyzed the demographic, clinical, and laboratory characteristics with descriptive statistic techniques. Descriptive statistics were stratified based on the presence or absence of perinephric fluid to assess group differences. We used the Shapiro–Wilk test to evaluate the distribution of the variables. Normally distributed continuous variables were reported as mean ± standard deviation and otherwise as the median and Q1–Q3. We reported categorical variables as absolute and relative frequencies. We performed comparisons using the Chi-square test with Yates correction or Fisher’s exact test for categorical variables and Student’s *t*-test or the Mann–Whitney’s U test, as appropriate, for continuous variables. We used no imputation techniques for the missing data. Patients with missing values in critical variables such as stone size, laboratory data (creatinine, leukocytosis, etc.), or imaging findings were excluded from relevant analyses. We used multivariate logistic regression models to assess the factors predicting the presence of the perinephric fluid collection. These models were adjusted for potential confounders such as age, sex, kidney stones or renal colic history, and baseline renal function. Effect sizes were communicated as odds ratios (ORs) or Beta coefficients, along with their 95% confidence intervals (CIs). A two-sided *p*-value < 0.05 was considered statistically significant. We reported this study following the Strengthening the Reporting of Observational Studies in Epidemiology (STROBE) statement [21] and presented the STROBE checklist in the Supplementary Material. We conducted all analyses using statistical software STATA/SE version 18 (StataCorp, College Station, TX, USA).

## 3. Results

**Figure 2:**
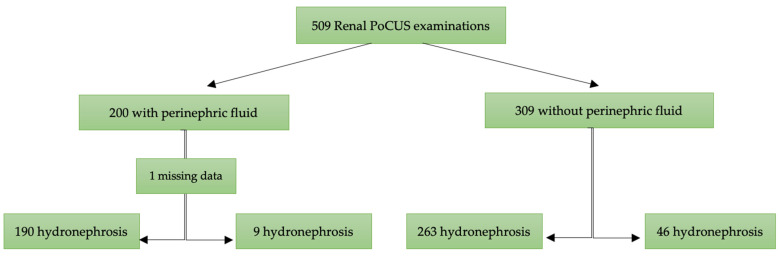
Flow chart of patients included for analysis. PoCUS: point-of-care ultrasound.

A total of 509 patients were included in this study, with 200 (39.3%) presenting with perinephric fluid and 309 (60.7%) without (Figure 2). The median age was 50 years (IQR: 41–60), with no significant difference between the two groups (*p* = 0.87). The proportion of males was higher in the group with perinephric fluid (72%) compared to those without (62%) (*p* = 0.02).

We have summarized the clinical and baseline characteristics of patients in Table 1. The two groups had no significant difference in the history of kidney stones or renal colic (*p* = 0.91). Hydronephrosis was present in 95% of patients with perinephric fluid compared to 85% of those without (*p* < 0.001). CT scans were more commonly performed in the perinephric fluid group (72% vs. 38%, *p* < 0.001). The stone position also varied between the groups, with distal ureter stones more frequent in the perinephric fluid group (64% vs. 55%, *p* = 0.001). There was no significant difference in stone size between the two groups (*p* = 0.35). The levels of creatinine and leukocytosis in the ED were also comparable.

The rate of urological interventions, including percutaneous nephrostomy and ureteral stent placement, was not significantly different between patients with or without perinephric fluid (*p* = 0.15 and *p* = 0.41, respectively).

Patients with hydronephrosis and perinephric fluid had urologic intervention 20.5% of the time, compared to 19.3% of patients with hydronephrosis and no perinephric fluid (*p* < 0.85).

We performed a sub-analysis of 94 patients with complete medical records requiring urological interventions to evaluate the association between perinephric fluid presence and clinical outcomes (Table 2). We found no statistically significant difference in terms of in-hospital complications, the incidence of sepsis, antibiotic, analgesic, and anti-inflammatory drug use, and the development of AKI and AKD.

In the multivariate logistic regression analysis (Table 3), considering the entire population, hydronephrosis and stone location were independently associated with the presence of perinephric fluid. Hydronephrosis significantly increased the odds of perinephric fluid presence (OR: 4.14; *p* = 0.007). Stone location also emerged as a significant factor, with stones in the proximal ureter (OR: 3.06; *p* = 0.003) and distal ureter (OR: 2.31; *p* = 0.018) more likely to be associated with perinephric fluid than kidney stones. Age, sex, fever, history of kidney stones, stone size, creatinine levels, and leukocytosis in the ED were not significantly associated.

## 4. Discussion

Perinephric fluid collection is a well-described pathological finding in the setting of acute ureteral stone obstruction. Our study found that perinephric fluid is a common occurrence in patients with renal colic and urolithiasis, with a prevalence of 39%. However, its presence does not significantly impact treatment decisions or clinical outcomes.

The prevalence we found is similar to the 46.5% reported by Nadav et al. [16] in their study on perinephric fluid among patients undergoing radiological US, particularly those with ureteral stones. However, it is higher than the 20% reported by Cannata et al. [15] in patients with ureterolithiasis identified by emergency physicians using POCUS. This discrepancy may be due to the focused nature of POCUS image acquisition with real-time interpretation, compared to more comprehensive US images collected by radiologists on high-resolution monitors.

The existing literature on the significance of perinephric fluid collection in the management of acute ureteral stone obstruction is lacking. Interestingly, despite its association with hydronephrosis (OR: 4.14, *p* = 0.007) and stones located in the proximal and distal ureters (OR: 3.06, *p* = 0.003; OR: 2.31, *p* = 0.018, respectively), perinephric fluid did not correlate with an increased need for urological intervention or prolonged hospitalization. This aligns with previous studies, such as that by Nadav et al., who found no significant relationship between perinephric fluid and the need for interventions [16]. Our sub-analysis of patients requiring urological interventions supports these findings, showing no statistically significant difference in in-hospital complications, sepsis rates, antibiotic use, AKI, or AKD between patients with and without sonographic perinephric fluid.

These results suggest that while perinephric fluid may indicate increased intrarenal pressure due to ureteral obstruction, it does not necessarily predict worse clinical outcomes. This contrasts with findings from Cannata et al., [15] who observed that in the setting of ureterolithiasis, perinephric fluid was associated with a stone size ≥ 5 mm (OR 4.00; *p* = 0.04) and a higher likelihood of requiring urological intervention (OR 10.38; *p* < 0.01) [15]. In their study, patients with hydronephrosis and perinephric fluid on POCUS underwent urological intervention 46.7% of the time, compared to just 4.3% of patients with hydronephrosis but no perinephric fluid (*p* < 0.01) [15]. To our knowledge, this is the first study to establish a relationship between perinephric fluid, stone size, and subsequent urological intervention. One possible explanation for this discrepancy is the difference in imaging modalities and clinical settings. POCUS, often performed by emergency physicians, may not provide the same level of detail as comprehensive US evaluations conducted by radiologists, which could lead to differences in the interpretation of findings.

Our data support the idea that perinephric fluid should not be a determining factor for immediate urological intervention. Even though patients with hydronephrosis and perinephric fluid underwent interventions 20.5% of the time, this rate was not significantly different from those with hydronephrosis alone (19.3%, *p* = 0.85). Furthermore, the length of hospital stay was comparable between patients with and without perinephric fluid (1.8 vs. 1.6 days, *p* = 0.40), reinforcing the conclusion that perinephric fluid does not necessitate more aggressive management and does not imply worse clinical outcomes for the patients.

Our results are in line with those of Nadav et al., who found that no patient outcome variables were significantly associated with the presence of perinephric fluid, except for more severe pain (OR 3.8 in the presence of any perinephric fluid, and OR 8.9 in cases of moderate-to-severe perinephric fluid) [16]. Chapman et al. and Setia et al. looked at non-US modalities and found a paradoxical decrease in complications associated with perinephric fluid or no association at all [22,23].

This study has several limitations that should be acknowledged. First, its retrospective and observational nature introduces inherent biases, including potential inaccuracies in data collection and limited control over confounding variables. As a single-center study, the results may not be generalizable to broader populations. Additionally, while we included a substantial number of patients, the relatively small sample size may have resulted in insufficient statistical power for some subgroup analyses, possibly leading to an underestimation of associations between perinephric fluid and certain clinical outcomes. Moreover, the need for standardized, validated, self-administered questionnaires to assess postoperative outcomes, such as pain intensity, limits our ability to evaluate patient-reported outcomes fully. The variability in diagnostic imaging may also have impacted the consistency of perinephric fluid detection, as image quality and operator expertise can vary across different settings. Lastly, while we focused on short-term clinical outcomes, such as the need for urological intervention and hospital length of stay, we did not include long-term follow-up to assess potential delayed complications of urolithiasis like renal abscess, sepsis, or chronic kidney disease. Conflicting reports exist regarding the rate of delayed complications related to perinephric fluid, such as renal abscess or sepsis, particularly in the context of nosocomial infections, and this warrants further investigation [24,25,26]. 

Further research is crucial to better understand the mechanisms behind perinephric fluid accumulation in acute urinary stone obstruction cases. Large-scale prospective studies are needed to explore the relationship between perinephric fluid collection and various clinical and laboratory parameters and to clarify the significance of this finding in managing acute cases in the ED. This study underscores the importance of collaboration between the radiologist’s US evaluation and the clinician’s assessment in the emergency setting.

## 5. Conclusions

In conclusion, our study highlights the presence of perinephric fluid as a common finding in patients with renal colic and urolithiasis, particularly in association with hydronephrosis and stone location in the proximal and distal ureter. However, despite its prevalence, the presence of perinephric fluid did not significantly correlate with the need for urological intervention, longer hospital stays, or worsening clinical outcomes. These findings suggest that while perinephric fluid may reflect increased pressure in the urinary tract, its clinical significance in determining management decisions remains uncertain. Further prospective research is necessary to better understand the implications of perinephric fluid in acute urolithiasis, particularly regarding potential delayed complications and long-term outcomes.

## Figures and Tables

**Table 1:** Clinical and baseline characteristics of patients with and without perinephric fluid. Values are median (Q1–Q3), means standard deviation, or numbers with percentages.

List of Variables	General Population	Perinephric Fluid Present	Perinephric Fluid Absent	*p* Value
Patient, n. (%)	509	200	309	
Age (years)	50 (41–60)	50 (41–62)	50 (40–59)	0.87
Sex, n. (%)				**0.02**
Male	337 (66)	145 (72)	192 (62)	
Female	172 (34)	55 (28)	117 (38)	
History of kidney stones or renal colic, n. (%)	202 (40)	79 (40)	123 (40)	0.91
Radiological exam, n. (%)				***p* < 0.001**
US	509 (100)	200 (100)	309 (100)	
CT	257 (50)	141 (72)	116 (38)	
Hydronephrosis, n. (%)				***p* < 0.001**
Present	453 (89)	190 (95)	263 (85)	
Absent	55 (11)	9 (5)	46 (15)	
Missing data	1 (0)	1 (0)	0	
Stone position, n. (%)				***p* = 0.001**
Kidney	98 (19)	20 (10)	78 (25)	
Proximal ureter	97 (19)	48 (24)	49 (16)	
Distal Ureter	299 (59)	128 (64)	171 (55)	
Bladder	10 (2)	4 (2)	6 (2)	
Missing data	5 (1)	5 (0)	5 (2)	
Stone size (mm)	6.77 ± 4.7	6.33 ± 3.5	7.06 ± 5.3	0.35
Fever in ED, n. (%)				0.76
Present	50 (10)	21 (11)	29 (9)	
Absent	452 (89)	175 (87)	277 (90)	
Missing data	7 (1)	4 (2)	3 (1)	
Creatinine in ED (mg/dL)	1.09 ± 0.5	1.14 ± 0.4	1.06 ± 0.6	0.21
Leukocytosis in ED (cells/µL)	10,064 ± 3883	10,865 ± 4648	9539 ± 3189	0.08
Surgical treatment	104 (20)	45 (22)	59 (19)	0.71
Percutaneous Nephrostomy	17 (3)	10 (5)	7 (2)	0.15
Ureteral JJ stent	22 (4)	11 (6)	11 (4)	0.41
URS during the same admission	32 (6)	14 (7)	18 (6)	0.72
Deferred URS	28 (6)	6 (3)	22 (7)	0.73
Missing data	5 (1)	4 (2)	1 (0)	
Medical expulsive therapy	405 (80)	155 (77)	250 (81)	0.41
Length of hospital stay (days)	1.6 ± 1.1	1.8 ± 1.1	1.6 ± 1	0.40

ED: emergency department; URS: Ureteroscopy. The bold used in the “*p*-value” column indicates a statistically significant *p*-value.

**Table 2:** Clinical outcomes in patients requiring urological interventions with and without sonographic perinephric fluid.

List of Variables	General Population	Perinephric Fluid Present	Perinephric Fluid Absent	*p* Value
Patient, n. (%)	94	40	54	
Complications within hospital stay, n. (%)	18 (19)	10 (25)	8 (15)	0.32
Clavien-Dindo classification of the complications within hospital stay, n. (%)				0.32
I–II	18 (19)	10 (25)	8 (15)	
III–V	0	0	0	
Sepsis, n. (%)	5 (5)	3 (8)	2 (4)	0.72
Antibiotics use, n. (%)	52 (55)	25 (63)	27 (50)	0.32
Analgesic use, n. (%)	58 (62)	26 (65)	32 (59)	0.72
Anti-inflammatory use, n. (%)	92	39 (98)	53 (98)	0.61
Acute kidney injury, n. (%)	4	2 (5)	2 (4)	0.83
Acute kidney disease, n. (%)	4	2 (5)	2 (4)	0.83

**Table 3:** Multivariate logistic regression analysis evaluating factors associated with perinephric fluid.

Variables	B	Odds Ratio	95% CI Lower	95% CI Upper	*p* Value
Age	0.14	1.014	1.000	1.029	0.052
Sex (Male)	−0.349	0.706	0.457	1.090	0.116
History of Kidney Stones at the same side	0.094	1.099	0.713	1.695	0.669
Hydronephrosis	1.421	4.141	1.472	11.650	**0.007**
Stone location (kidney)					0.012
Stone location (proximal ureter)	1.118	3.059	1.459	6.414	**0.003**
Stone location (distal ureter)	0.839	2.314	1.154	4.641	**0.018**
DT stone (<5 mm)					0.204
DT stone (5–10 mm)	−0.405	0.667	0.424	1.048	0.079
DT stone (>10 mm)	−0.428	0.652	0.283	1.503	0.315
Fever in ED	−0.046	0.955	0.497	1.834	0.891
Creatinine in ED	0.002	1.002	0.998	1.005	0.283
Leukocytosis in ED	0.000	1.000	1.000	1.001	0.678

ED: emergency department. The bold used in the “*p*-value” column indicates a statistically significant *p*-value.

## Data Availability

Data presented in this study are available on request from the corresponding authors.

## Supplementary Materials

The following supporting information can be downloaded at: https://www.mdpi.com/article/10.3390/jcm13206118/s1, STROBE Statement—checklist of items that should be included in reports of observational studies.
